# Miscellany

**Published:** 1888-04

**Authors:** 


### Miscellany.

PERIODS OF ISOLATION FOR ERUPTIVE FEVERS.—Ollivier, in a report read to *Acad. de Med.*, advises that school children attacked with small-pox be strictly isolated for forty days, counting from the first day of the invasion. The same period should be observed for scarlet fever and diphtheria, while twenty-five days suffice for varicella, measles and mumps. Before the period of isolation has expired, the child should have several baths with soap, his clothing fumigated, the chamber well aired, and before being readmitted to the school, the physician should certify that these precautions have been carried out.

A free discussion followed upon the time necessary for whooping-cough, but no conclusion was reached.—*Medical Times.*

---

WHEN it is a question of nerves, the power of imagination is supposed to be stronger in women than in men, but this was not shown in a recent hospital experiment. Dr. Durand, wishing to test the practical effect of mind disease, gave a hundred patients a dose of sweetened water. Fifteen minutes after, entering apparently in great excitement, he announced that he had, by mistake, given a powerful emetic, and preparations must be made accordingly. Eighty out of the hundred patients became thoroughly ill, and exhibited the usual result of an emetic; twenty were unaffected. The curious part of it is that, with very few exceptions, the eighty "emeticised" subjects were men, while the strong-nerved few, who were not to be caught with chaff, were women.—*Medical and Surgical Reporter.*

---

"DOCTOR" is a very promiscuous title in America. The preacher is a "doctor." The school principal is a "doctor." The family physician is a "doctor." The veterinary surgeon is a "doctor." The dentist is a "doctor." The manufacturer of patent medicines is a "doctor." The remover of pedal excrescences, "without pain," is a "doctor." And so on. There are almost as many "doctors" in the South. But this is a free country. In Germany, under the despotism of a monarchy, this freedom is not allowed. An American dentist has just been fined in Berlin for putting "Doctor" on his cards.—*Medical and Surgical Reporter.*

The Use of Bedbugs, Fleas and Lice.—A Rochester quack, in a pretentious address to the public, has disposed of a question which has long vexed the minds of thinking men. He addresses the profession in Latin, beginning, "*Quod inscripsi, erores artis Hypocraticae*," etc., but soon, very fortunately, begins in English to tell of his wonderful discoveries, among which may be mentioned the use of vermin. We quote from his brochure: "But the most wonderful thing as a hygienic safeguard, in the opinion of the philosopher, Nature supplies in the creation of the louse, bed-bug, flea and fly. The lazy, uncleanly and filthy are thereby constrained to scratch themselves, to the end that the purification and renovation of the skin, the combustion and moulting, so to speak, may properly go on. For precisely the same reason, is the inert phlegmatic restrained from his prolonged nap in the heat of summer by an industrious fly, and how ingraciously he thanks the preserver of his life—the fly who disturbs him in his slumber! Alas! ingratitude is the reward of the world. And again the bed-bug—to what superhuman exertions in the interests of cleanliness is the busy housewife constrained, and to what exemplary diligence! Aye, indeed, the bed-bug is the unrecognized guardian—the faithful sentinel of Hygea. For the New Jersey mosquito, no knight ventures to lay a lance in rest; whilst the usefulness of the fly is incontestable. The time will soon come in which we may observe their restless flight, in seemingly purposeless gyrations. But they purify the air by a consumption of the bacteries, etc.; for just as the bees carry a powder upon their legs, do the flies carry a viscous substance upon theirs, for the removal of the invisible but obnoxious vermin."

---

Forced Respiration without Tracheotomy.—The attention of our readers has recently been attracted to Dr. George Fell's cases of opium poisoning treated by forced respiration with preliminary tracheotomy. Dr. Fell was probably the first to employ forced respiration with the bellows in the treatment of opium poisoning. In discussing apnœa in general, Ashurst (Principles and Practice of Surgery, page 370, edition of 1885,) mentions, among other methods of artificial respiration, that "*inflation with the bellows*, provided with a suitable mouth-piece or nose-piece, may be efficiently used, provided that care be taken to secure expiration by manual compression, and that the instrument be worked gently and not more than ten or twelve times in a minute." Also on page 375, in discussing apnœa after tracheotomy, he states: "Dr. Beverly Robinson, of New York, has devised an ingenious "insufflator," by means of which the surgeon can directly inflate the patient's chest through the tracheal tube. This intrument, or a *double-acting bellows*, as recommended by Dr. B. W. Richardson, should be employed in case suffocation is threatened. If neither of these be at hand, an ordinary hand-ball syringe (reversed) may be used, as suggested by Dr. Green, of Brooklyn." From these statements, we are forced to believe that Dr. Fell's method or operation is not new, and that tracheotomy is not necessary in the treatment of opium apnœa.

---

Of the 582 new students enrolled in the School of Medicine, University of Paris, this year, 479 were French and 103 foreigners. Of the foreigners, twenty are Americans, twenty Servians, eleven Roumanians and eleven Turks. There are eleven women among the new students, and it is somewhat remarkable that all are foreigners, ten being Russians and one Greek. During and after the rule of the Commune in 1871, there were many French women who desired to obtain equal educational advantages with men. They were successful; many began the study of medicine and law, while some, as in the previous revolutions, dabbled in politics. Now that they have succeeded in obtaining their "rights," they seem perfectly contented to let the world go on in accordance with the old order of things, leaving the enslaved women of Russia and the United States to struggle for liberty and equal rights with men.

---

Student-Life in Switzerland.—The students of the University of Berne recently sent a challenge to those of Zurich. The Tigurin Corps immediately responded by sending twenty of their best swordsmen. The duels continued until night, and were finished with a parade. When the train departed for Zurich, fifteen carriages in procession conveyed the wounded to the depot. Our French exchange, *Le Progres Medical*, regrets that two of the students took advantage of this dueling tournament to settle some old quarrels, and as a result one was very seriously wounded.

Dr. A. W. Abbott publishes, in the *North-western Lancet*, what appears to be a treatise on the art of defecation. He attributes to the ordinary method of performing this operation on a seat, a number of serious ills, such as constipation, hemorrhoids, uterine displacements, and recommends a return to the primitive custom of squatting, as practiced by our uncivilized ancestors. It is to be hoped that some genito-urinary specialist will soon describe the best method of making water.

---

Try the following prescription to abort an attack of acute bronchitis. Prof. H. C. Wood says that it is worth $5,000 to every medical student:

| | | |
|---|---|---|
| ℞ | Potassii citratis . . . . . . . . . . . . . . . . . | ℥ j |
| | Syrupi ipecacuanhæ. . . . . . . . . . . . . . . | f ℥ j |
| | Succi limonis . . . . . . . . . . . . . . . . . . . | f ℥ ij |
| | Aquæ. . . . . . . . . . . . . . . . . . . . . . | ℥ iij |

M. S.—Two teaspoonfuls every three hours.—*Medical Times.*

---

We acknowledge the receipt of No. 1, Vol. I., of the *Climatologist*, published in Washington, and conducted by Wm. C. Chace. This new journal is devoted specially to climatotherapy, medical geography, demography, preventive medicine, etc. The journal is published quarterly, and the subscription price fifty cents a year. These branches of medical science are growing in importance, and we predict that the *Climatologist* will successfully fill a place hitherto vacant in medical literature.

---

Chagres Fever at Panama.—It would be difficult to find a more unhealthy place than the Panama Canal at present. The hospitals are overcrowded, and many of the poorer workmen die in their tents, and even at their work. At Bas Obispo, with a population of 1,000, the death-rate is five or six a day, and sometimes reaches as high as twenty.

---

Dr. F. R. Drake, Clinical Professor of Medicine, University of New York, died at his home March 1st, after a brief illness. Dr. Drake was in the prime of his life and professional career, being only forty-three years of age.

Another long-felt Want.—Caller—" Any back numbers of your magazine?"

Health Journal Editor—" Yes, sir. Which number do you wish?"

"I do not know the date, but I saw a line in a paper to the effect that it had an article entitled, 'How to lie when asleep.'"

"I know which number you want. Having trouble at night, eh?"

"I should say so. My wife says I talk in my sleep, and I know from the way she acts that I tell the truth."

---

A London lady, who was a chronic invalid, after many years' treatment without improvement, was finally notified by her physician that he had exhausted all the resources of his art, and that, in his opinion, *tempus edax rerum*, was the only remedy which would help her. Not being a Latin scholar, she applied for this remedy at her chemist's, and he put up a compound which she continued to take for some years. The fraud was finally detected, and now a suit for damages against the chemist is in progress.

---

It is claimed that the opium habit can be successfully counteracted by the substitution of hypodermic injections of antipyrin. The antipyrin, it is said, acts as a sedative to the nervous system, but has no narcotic properties; hence, it can be used an indefinite time without causing the patient to contract the antipyrin habit.

---

The New York State Legislature has appropriated $185,000 for the erection of a new State Asylum for Insane Criminals, to be located at Fishkill. There will be a farm of 246 acres. The buildings are to be on a pavilion plan. There will be six general pavilions, two infirmaries, and a central administration building.

---

A young doctor, who had his eye on a banker's daughter, thought to win the favor of the father, who was his patient, by sending him a very low bill. Soon afterwards, he asked the father for the daughter's hand. To his surprise, the father refused, and, being pressed for his reason for refusing, told the young man he could not trust his daughter to a man who had such a poor head for accounts.—*Fliegende Blätter*.

A LITTLE boy came to a druggist with a prescription for castor-oil. The druggist put it up, and the boy offered ten cents in payment. "That is not enough money," said the druggist. "There's fifteen cents' worth in that bottle." The boy thought awhile, and then answered: "I'll tell you. You drink five cents' worth, and then the money will be enough."—*Fliegende Blätter.*

---

IN the United States, annually, the sales of patent medicines amount to $22,000,000, of which $5,000,000 is profit, and $10,000,000 is paid for advertising. These figures suggest a reason why so many druggists prepare a "cough mixture" from a private formula. It is, in fact, oftentimes a patent medicine in embryo.

---

DR. FREDERICK PETERSON, formerly of this city, and recently connected with the Poughkeepsie State Insane Asylum, has removed to New York City, and opened an office at 34 East Twenty-first street.

---

"I AM sorry," said a clergyman, "that your wife has such a heavy burden to bear." "Yes, she does—that's so," acquiesced his parishioner. "She's laid flat on her back this seven years. Seems as if she'd get altogether worn out. I do wish she'd get well—or something."

---

DR. FOWLER BRADNACK, of this city, has recently published an amusing satire on quack advertising, entitled, "Dr. Case's Handbill." It may be obtained at McCready's.

---

The Medical Department of the Western Reserve University, Cleveland, has announced that hereafter a three years' course will be obligatory, and a matriculation examination required.

---

SALOL, although given a faithful trial in acute rheumatic cases, does not give as good satisfaction as salicylate of sodium, says the *Medical Times.*

---

A CALIFORNIA court has decided that a private hospital, in a populous neighborhood, is a nuisance, and must be removed.

MARKED pulsation at the supra-sternal notch and over the innominate, in aortic insufficiency, should not be mistaken for aneurism. The beat is not expansile, as in aneurism.—*Osler.*

---

WHEN convulsions first occur after the thirtieth year, and usually epileptiform in character, suspicion points to cerebral tumor.—*Osler.*

---

ONE hundred and forty-three students were graduated at the recent commencement of Bellevue Hospital Medical College.

---

CHILLS and fever, intermittent high temperature, and pus in the urine, the urine being acid, point to pyelitis.—*Osler.*

---

PROF. R. A. PENROSE has resigned from the faculty of the Medical Department of the University of Pennsylvania.

---

IN Paris, a Stomatological Society has been founded. The object is the study of diseases of the mouth.

---

IN abscess of the brain, the pus is often acid in reaction. The cause is not known.—*Tyson.*

---

DROPSY does not occur in mitral insufficiency, unless tricuspid insufficiency co-exists.—*Osler.*

